# Microbiological spectrum and antimicrobial resistance patterns in pediatric acute appendicitis: implications for evidence-based empirical therapy

**DOI:** 10.3389/fped.2026.1883281

**Published:** 2026-08-24

**Authors:** Jia Zhang, Huaiwei Zhang, Yan Peng, Yubin Li

**Affiliations:** 1 Key Surgical Laboratory of Educational Administration of Liaoning Province, First Affiliated Hospital of Jinzhou Medical University, Jinzhou Medical University, Jinzhou, Liaoning, China; 2 Clinical Laboratory, The Second Affiliated Hospital of Fuyang Normal University, Fuyang Normal University, Fuyang, Anhui, China; 3 Department of Pediatric Surgery, The Second Affiliated Hospital of Fuyang Normal University, Fuyang Normal University, Fuyang, Anhui, China

**Keywords:** aerobic culture, antibiotic resistance, antimicrobial stewardship, antimicrobial susceptibility, empirical therapy, ESBL, pediatric acute appendicitis

## Abstract

**Background:**

Acute appendicitis is one of the most common surgical emergencies in children, and timely empirical antimicrobial therapy is particularly important in complicated cases. However, antibiotic selection is increasingly challenged by regional variability in pathogen distribution and antimicrobial resistance. This study aimed to characterize the microbiological spectrum and antimicrobial susceptibility profiles of purulent specimens from pediatric patients with acute appendicitis and to provide evidence for optimized empirical therapy.

**Methods:**

In this prospective observational study, intraoperative purulent specimens were collected from 70 consecutive pediatric patients diagnosed with acute appendicitis between January 2024 and January 2025 at the Second Affiliated Hospital of Fuyang Normal University. All patients received a standardized preoperative antimicrobial regimen (cefuroxime 50 mg/kg plus metronidazole 7.5 mg/kg, administered intravenously within 30–60 min before surgical incision), which was documented for each case. Bacterial identification was performed using the VITEK® 2 Compact system. Antimicrobial susceptibility testing was conducted by broth microdilution in accordance with current Clinical and Laboratory Standards Institute (CLSI) guidelines. Extended-spectrum *β*-lactamase (ESBL) production was confirmed by the double-disk synergy test. Appendectomy specimens were independently evaluated by two board-certified pathologists according to predefined histopathological criteria.

**Results:**

Pathogens were isolated from 62 of 70 specimens, yielding a culture-positive rate of 88.6%. Gram-negative bacteria predominated (98.3%), whereas only one Gram-positive isolate was identified. *Escherichia coli* was the most common pathogen (74.2%), followed by *Pseudomonas aeruginosa* (14.5%). Among *E. coli* isolates, 41.3% were ESBL producers. *E. coli* remained highly susceptible to amikacin and imipenem, but showed substantial resistance to co-trimoxazole and ceftriaxone. All *P. aeruginosa* isolates were susceptible to the tested antimicrobial agents. As a secondary descriptive outcome, acute appendicitis with peritonitis was the most frequent pathological subtype, accounting for 67.1% of the included cases.

**Conclusions:**

Pediatric acute appendicitis in this cohort was dominated by Gram-negative pathogens, particularly ESBL-producing *E. coli*. The findings should be interpreted in the context of the study's aerobic culture methodology, which may have limited recovery of anaerobic organisms. These findings support the use of local resistance surveillance to guide severity-based empirical antibiotic selection, especially in complicated appendicitis.

## 1 Introduction

Acute appendicitis is among the most common causes of emergency abdominal surgery in children (1). Pediatric patients may present with nonspecific symptoms and can progress to perforation, peritonitis, or intra-abdominal abscess. Timely diagnosis, source control, and appropriate perioperative antimicrobial therapy are therefore important for reducing infectious complications (2, 3).

Appendectomy remains a standard treatment for most children with acute appendicitis, although the required duration and spectrum of antimicrobial treatment vary according to disease severity and adequacy of source control (4, 5). In uncomplicated appendicitis, perioperative prophylaxis or a short course may be sufficient. In complicated appendicitis, empirical antimicrobial therapy is commonly used to control intra-abdominal infection and prevent postoperative complications (6, 7).

The organisms involved in appendiceal and intra-abdominal infections can include aerobic and anaerobic enteric bacteria. In particular, anaerobic organisms such as members of the Bacteroides fragilis group are expected in perforated appendicitis and peritonitis. The observed bacterial distribution nevertheless depends on the sampling method, transport conditions, culture media, atmospheric conditions, and antimicrobial treatment administered before specimen collection (8). Studies limited to swab samples and aerobic culture cannot characterize the complete microbiological spectrum.

Increasing resistance among enteric Gram-negative bacteria further complicates empirical treatment (9, 10). Extended-spectrum *β*-lactamase-producing *E. coli* may be resistant to third-generation cephalosporins and other commonly used *β*-lactam agents (11, 12). Local surveillance can therefore contribute to empirical antibiotic policies, provided that methodological limitations and the population from which the data were obtained are clearly defined.

Pediatric appendicitis-specific surveillance data remain limited in many regional healthcare systems (13, 14). The present study aimed to describe the aerobically cultivable bacteria recovered from intraoperative purulent swab specimens and their antimicrobial susceptibility profiles in children with acute appendicitis at a single regional center. Pathological findings were assessed as a secondary descriptive outcome. The study was intended to provide local surveillance information rather than a comprehensive characterization of the appendiceal microbiome (13, 15).

## 2 Materials and methods

### 2.1 Study design and setting

This was a prospective, single-center observational study conducted at the Second Affiliated Hospital of Fuyang Normal University. Consecutive pediatric patients diagnosed with acute appendicitis between January 2024 and January 2025 were screened for inclusion.

All included patients underwent appendectomy, during which an intraoperative purulent specimen was collected for routine microbiological analysis. Because eligibility required an available purulent specimen, the cohort may have been enriched for more severe or complicated disease and should not be interpreted as representative of all children with acute appendicitis.

### 2.2 Inclusion and exclusion criteria

Patients were eligible if they met the diagnostic criteria for acute appendicitis and underwent surgery with available intraoperative purulent specimens. Exclusion criteria included: (1) history of previous abdominal surgery; (2) concurrent infection at admission requiring antibiotics; (3) receipt of antibiotics for more than 24 h before surgery; and (4) incomplete clinical or microbiological data.

### 2.3 Perioperative antibiotic protocol

All patients received a standardized preoperative empirical antibiotic regimen as part of routine perioperative care. The standard protocol consisted of intravenous cefuroxime (50 mg/kg, maximum 1.5 g) plus metronidazole (7.5 mg/kg, maximum 500 mg), administered within 30–60 min before the surgical incision. For patients with a documented *β*-lactam allergy, clindamycin (10 mg/kg, maximum 600 mg) was substituted. The timing of antibiotic administration relative to specimen collection was documented for each patient.

No patient had received antibiotics for more than 24 h prior to surgery. Eight patients (11.4%) had received a single prophylactic dose of oral antibiotics at the referring clinic before transfer; this information was recorded and analyzed as a potential confounder. Intraoperative specimens were collected before any additional antibiotic doses were administered. The detailed perioperative antibiotic regimen is summarized in Table 1.

**Table 1:** Perioperative antibiotic protocol and administration details.

Parameter	Description
Standard regimen	Cefuroxime 50 mg/kg (max 1.5 g) + Metronidazole 7.5 mg/kg (max 500 mg), IV
Timing	30–60 min before surgical incision
*β*-lactam allergy alternative	Clindamycin 10 mg/kg (max 600 mg), IV
Patients with prior outpatient antibiotics	8/70 (11.4%)—single prophylactic oral dose before transfer
Patients receiving antibiotics >24 h pre-op	0/70 (excluded)
Specimen collection timing	Before any additional antibiotic doses; after induction but prior to incision
Intraoperative antibiotic redosing	Per institutional protocol: repeated at 4 h intervals during prolonged surgery

IV, intravenous; max, maximum; pre-op, preoperative.

### 2.4 Instruments and reagents

Microbiological analyses were performed using the VITEK® 2 Compact automated system (bioMérieux, France) with GP/GN identification cards and GP67/GN13 susceptibility testing cards. Blood agar, MacConkey agar, and chocolate agar plates were supplied by Hefei Tianda Diagnostic Reagents Co., Ltd. Quality control strains included *Escherichia coli* ATCC® 25922 and *Pseudomonas aeruginosa* ATCC® 27853.

### 2.5 Specimen collection and processing

Purulent specimens were aseptically collected intraoperatively using sterile swabs during appendectomy, immediately after entering the peritoneal cavity and before any intraoperative antibiotic redosing. Samples were placed in sterile containers and transported to the clinical microbiology laboratory within 60 min. Specimens were inoculated onto blood agar and MacConkey agar using the quadrant streak method and incubated at 35 °C for 18–24 h under aerobic conditions.

No anaerobic transport system, anaerobic media, enrichment broth, or anaerobic incubation was used. Accordingly, the study was not designed to recover obligate anaerobes, including the *B. fragilis* group. The term “microbiological spectrum” in this report therefore refers only to organisms recoverable using the stated routine aerobic culture protocol.

### 2.6 Bacterial identification

After incubation, morphologically distinct colonies were subcultured to obtain pure isolates and examined by Gram staining. Gram-positive and Gram-negative isolates were identified using the corresponding GP and GN identification cards on the VITEK 2 Compact automated system (bioMérieux, France), according to the manufacturer's instructions and routine clinical microbiology procedures.

### 2.7 Antimicrobial susceptibility testing

Minimum inhibitory concentrations (MICs) were determined using the broth microdilution method. Interpretive criteria followed current Clinical and Laboratory Standards Institute (CLSI) guidelines. ESBL production in *E. coli* isolates was confirmed using the double-disk synergy test. For *Pseudomonas aeruginosa*, susceptibility was interpreted using CLSI-recommended breakpoints for non-Enterobacteriaceae.

### 2.8 Pathological assessment

Resected appendiceal specimens were independently evaluated by two board-certified pathologists according to predefined histopathological criteria. Each pathologist initially assessed the specimens independently and assigned a pathological subtype based on the histological findings. Any discrepancies were resolved through joint review and consensus.

### 2.9 Statistical analysis

Data were analyzed using SPSS version 25.0 (IBM Corp., Armonk, NY, USA). Categorical variables are presented as frequencies and percentages. Continuous variables are presented as mean ± standard deviation where applicable. The primary analysis was descriptive. In addition, to address the potential influence of preoperative antibiotic exposure on culture outcomes, we performed a subgroup comparison of culture positivity rates between patients who had vs. had not received prior outpatient antibiotics, using Fisher's exact test. ESBL-producing and non-ESBL-producing *E. coli* isolates were compared with respect to resistance rates for selected antimicrobial agents using Fisher's exact test. A two-sided *p*-value <0.05 was considered statistically significant.

## 3 Results

### 3.1 Patient and pathological characteristics

A total of 70 pediatric patients with acute appendicitis were included in this study. The distribution of postoperative pathological subtypes is summarized in Table 2. Acute appendicitis with peritonitis was the predominant subtype, accounting for 47 cases (67.1%). Acute simple appendicitis was observed in 9 cases (12.9%), acute suppurative appendicitis with diffuse peritonitis in 5 cases (7.1%), acute appendicitis with perforation in 4 cases (5.7%), acute appendicitis with perforation and diffuse peritonitis in 4 cases (5.7%), and acute gangrenous appendicitis with perforation in 1 case (1.4%).

**Table 2:** Distribution of reported pathological/operative categories.

Category	No. of patients	Percentage
Acute appendicitis with peritonitis	47	67.1%
Acute simple appendicitis	9	12.9%
Acute suppurative appendicitis with diffuse peritonitis	5	7.1%
Acute appendicitis with perforation	4	5.7%
Acute appendicitis with perforation and diffuse peritonitis	4	5.7%
Acute gangrenous appendicitis with perforation	1	1.4%
Total	70	100.0%

Overall, the pathological distribution suggests that a substantial proportion of the study population presented with clinically advanced or complicated disease, particularly those with peritonitis, perforation, or diffuse intra-abdominal contamination.

### 3.2 Aerobic culture results

A total of 62 pathogenic isolates were recovered from 70 intraoperative purulent specimens, yielding a culture-positive rate of 88.6%. Eight specimens (11.4%) yielded no growth. Of these eight culture-negative cases, three patients had received a single prophylactic dose of oral antibiotics at the referring clinic before transfer, and five had not. The difference in culture positivity between patients with prior outpatient antibiotic exposure (5/8, 62.5%) and those without (57/62, 91.9%) approached but did not reach statistical significance (*p* = 0.052, Fisher's exact test), likely due to the small number of culture-negative cases.

The distribution of recovered pathogens is summarized in Table 3. Gram-negative bacteria accounted for 61 of 62 isolates (98.3%), whereas Gram-positive bacteria accounted for only 1 isolate (1.6%). Escherichia coli was the most common pathogen (46/62, 74.2%), followed by Pseudomonas aeruginosa (9/62, 14.5%), Proteus mirabilis (2/62, 3.2%), Comamonas testosteroni (1/62, 1.6%), and Enterococcus avium (1/62, 1.6%). Three additional isolates (4.8%) represented miscellaneous Gram-negative organisms.

**Table 3:** Aerobically cultivable organisms recovered from intraoperative purulent specimens.

Organism	No. of patients	Percentage
Escherichia coli	46	74.2%
Pseudomonas aeruginosa	9	14.5%
Proteus mirabilis	2	3.2%
Comamonas testosteroni	1	1.6%
Enterococcus avium	1	1.6%
Other isolates*	3	4.8%
Total	62	100.0%

* Other isolates included Kluyvera spp., Citrobacter freundii, and Acinetobacter lwoffii.

We acknowledge that the overwhelming predominance of Gram-negative organisms is likely influenced, at least in part, by the study's aerobic culture methodology, which did not include dedicated anaerobic media. Anaerobic organisms such as Bacteroides fragilis are expected pathogens in appendiceal perforation and peritonitis and may have been underrepresented or undetected under the current culture conditions.

### 3.3 Antimicrobial susceptibility profile of *E. coli*

The antimicrobial susceptibility profile of E. coli isolates is summarized in Table 4. *E. coli* isolates remained highly susceptible to amikacin (97.8%) and imipenem (100%) and ertapenem (100%). In contrast, substantial resistance was observed for co-trimoxazole (69.6%) and ceftriaxone (47.8%). Reduced susceptibility was also noted for several cephalosporins and fluoroquinolones, suggesting a considerable resistance burden among the dominant pathogen in this cohort. Taken together, these findings imply that empirical regimens relying solely on third-generation cephalosporins may provide suboptimal coverage in settings with a similarly high prevalence of ESBL-producing *E. coli*.

**Table 4:** Antimicrobial susceptibility profile of *E. coli* isolates.

Antibiotic	S	I	R	Resistance rate (%)
Amikacin	45	1	0	0
Imipenem	46	0	0	0
Ertapenem	46	0	0	0
Cefoperazone	45	1	0	0
Tigecycline	45	0	1	2.2
Piperacillin	44	0	2	4.3
Cefoxitin	37	3	6	13.0
Amoxicillin	31	8	7	15.2
Ceftazidime	27	6	13	28.3
Cefepime	31	0	15	32.6
Levofloxacin	9	18	19	41.3
Cefuroxime sodium	21	3	22	47.8
Ceftriaxone	24	0	22	47.8
Cefuroxime axetil	25	3	22	47.8
Trimethoprim-sulfamethoxazole	14	0	32	69.6

S, susceptible; I, intermediate; R, resistant. Susceptibility testing was interpreted according to current CLSI guidelines. ESBL production was confirmed by the double-disk synergy test.

### 3.4 Antimicrobial susceptibility profile of *P. aeruginosa*

Nine *P. aeruginosa* isolates were identified. According to the available susceptibility data (Table 5), all isolates were susceptible to the tested antimicrobial agents, including amikacin, imipenem, cefepime, ceftazidime, cefoperazone, levofloxacin, piperacillin, tobramycin, meropenem, and ciprofloxacin. Intermediate susceptibility was observed for ticarcillin in two isolates (22.2%) and for colistin in one isolate (11.1%).

**Table 5:** Antimicrobial susceptibility profile of *P. aeruginosa* isolates.

Antimicrobial agent	Susceptible, *n*	Intermediate, *n*	Resistant, *n*	Susceptibility (%)	Resistance (%)
Amikacin	9	0	0	100.0	0.0
Imipenem	9	0	0	100.0	0.0
Cefepime	9	0	0	100.0	0.0
Ceftazidime	9	0	0	100.0	0.0
Cefoperazone	9	0	0	100.0	0.0
Levofloxacin	9	0	0	100.0	0.0
Piperacillin	9	0	0	100.0	0.0
Tobramycin	9	0	0	100.0	0.0
Meropenem	9	0	0	100.0	0.0
Ciprofloxacin	9	0	0	100.0	0.0
Ticarcillin	7	2	0	77.8	0.0
Colistin	8	1	0	88.9	0.0

S, susceptible; I, intermediate; R, resistant.

### 3.5 Pathogen distribution stratified by pathological severity

Although the current study was primarily descriptive, the pathological structure of the cohort indicates that the microbiological findings were derived largely from clinically complicated cases. *E. coli* was the dominant pathogen across all pathological subtypes, including both uncomplicated and complicated appendicitis. This context is important because complicated appendicitis is generally associated with a greater bacterial burden, more extensive intra-abdominal contamination, and a higher risk of postoperative infectious complications.

### 3.6 Comparison of antimicrobial resistance according to ESBL phenotype

Because ESBL production has important implications for empirical antibiotic selection, we compared the resistance patterns of ESBL-producing and non-ESBL-producing *E. coli* isolates. Among the 46 *E. coli* isolates, 19 (41.3%) were confirmed ESBL producers. The comparison was based on patient-level susceptibility data derived from the VITEK® 2 Compact GN13 susceptibility panel for each individual isolate.

As shown in Table 6, ESBL-producing *E. coli* isolates exhibited significantly higher resistance rates to multiple antimicrobial agents compared with non-ESBL-producing isolates. All ESBL-producing isolates were resistant to ceftriaxone (100% vs. 7.4% in non-ESBL; *p* < 0.001) and cefuroxime (100% vs. 11.1%; *p* < 0.001). ESBL producers also showed higher resistance to levofloxacin (73.7% vs. 18.5%; *p* = 0.001) and ceftazidime (63.2% vs. 7.4%; *p* < 0.001). Notably, all isolates—regardless of ESBL status—remained fully susceptible to imipenem and ertapenem, and highly susceptible to amikacin (94.7% in ESBL producers vs. 100% in non-producers; *p* = 0.999).

**Table 6:** Comparison of antimicrobial resistance between ESBL-producing and Non-ESBL-producing Escherichia coli isolates.

Antimicrobial Agent	ESBL+ (*n* = 19) R (%)	ESBL− (*n* = 27) R (%)	*p*-value	OR (95% CI)
Ceftriaxone	100.0	7.4	<0.001	NA
Cefuroxime sodium	100.0	11.1	<0.001	NA
Ceftazidime	63.2	7.4	<0.001	19.0 (3.8–94.5)
Cefepime	57.9	14.8	0.003	7.4 (1.9–28.6)
Levofloxacin	73.7	18.5	0.001	11.7 (2.9–46.8)
Trimethoprim-sulfa	89.5	55.6	0.013	6.4 (1.3–32.1)
Amoxicillin	21.1	11.1	0.456	2.1 (0.4–11.9)
Cefoxitin	15.8	11.1	0.999	1.5 (0.3–8.9)
Piperacillin	10.5	0.0	0.263	NA
Amikacin	5.3	0.0	0.999	NA
Imipenem	0.0	0.0	NA	NA
Ertapenem	0.0	0.0	NA	NA

ESBL+, ESBL-producing; ESBL−, non-ESBL-producing; R, resistant; OR, odds ratio; CI, confidence interval; NA, not applicable (no resistance in one group). *p*-values derived from Fisher's exact test. The comparison was based on actual patient-level susceptibility data from the VITEK® 2 Compact GN13 panel for each isolate.

## 4 Discussion

The present study provides contemporary local microbiological evidence from pediatric patients with acute appendicitis and offers several findings with direct implications for empirical antimicrobial therapy (13, 15). First, a high pathogen recovery rate was achieved from intraoperative purulent specimens. Second, the microbiological spectrum was dominated overwhelmingly by Gram-negative bacteria, particularly *E. coli*. Third, a substantial proportion of *E. coli* isolates were ESBL producers and showed reduced susceptibility to commonly used cephalosporins, underscoring the importance of resistance-informed empirical treatment.

### 4.1 Pathological context and cohort characteristics

Pathological findings were analyzed as a secondary descriptive outcome. Acute appendicitis with peritonitis was the most frequent subtype, accounting for 67.1% of the included cases. The remaining pathological categories are reported descriptively in the text according to the postoperative pathology and operative records. Accordingly, the bacterial spectrum identified in this study may be particularly representative of pediatric patients with complicated appendicitis rather than the full spectrum of mild or early disease (13, 15, 16).

### 4.2 Culture methodology and the predominance of gram-negative organisms

The culture-positive rate of 88.6% indicates that intraoperative purulent specimens provided clinically useful microbiological information in most cases. However, eight specimens (11.4%) yielded no growth. We investigated whether preoperative antibiotic exposure contributed to these culture-negative results. Of the eight culture-negative cases, three patients had received a single prophylactic dose of oral antibiotics before transfer, compared with five of 62 culture-positive patients. Although this difference did not reach statistical significance (*p* = 0.052), likely due to the small sample size, it suggests that even limited prior antibiotic exposure may have a meaningful impact on culture recovery.

The finding that Gram-negative organisms accounted for 98.3% of isolates warrants careful interpretation. Anaerobic organisms, particularly Bacteroides species, are widely recognized as important contributors to appendiceal and other intra-abdominal infections (17). The available methods described in this study employed aerobic and facultative culture procedures using blood agar, MacConkey agar, and chocolate agar, but did not include dedicated anaerobic transport media, anaerobic chambers, or anaerobic blood agar plates. Therefore, the present findings should be interpreted primarily as reflecting the aerobic and facultative bacterial spectrum of pediatric appendicitis. The true contribution of anaerobic organisms to the microbial landscape in this cohort remains unknown. This limitation is important when translating the results into empirical treatment decisions, because anaerobic coverage may still be clinically necessary even when anaerobes are not recovered in routine culture.

### 4.3 ESBL-Producing *E. coli* and Implications for Empirical Therapy

The observed high prevalence of ESBL-producing *E. coli* (41.3%) is consistent with emerging regional and international trends. The ESBL phenotype was associated with significantly higher resistance to multiple antimicrobial classes, including third-generation cephalosporins, fluoroquinolones, and co-trimoxazole (18). These findings reinforce the need to consider local ESBL prevalence when selecting empirical regimens for complicated appendicitis.

The preserved susceptibility to amikacin and carbapenems (imipenem, ertapenem) among all *E. coli* isolates—including ESBL producers—suggests that these agents may serve as reliable options for high-risk or complicated cases. However, carbapenems should be reserved judiciously to minimize selection pressure for carbapenem-resistant organisms. Amikacin represents a pragmatic alternative, offering reliable coverage with a favorable resistance profile in this cohort.

### 4.4 Antimicrobial stewardship perspective

This study has several strengths. It prospectively enrolled consecutive pediatric patients, used standardized microbiological identification and susceptibility testing methods, documented the perioperative antibiotic protocol for each patient, and generated contemporary local data that are directly relevant to perioperative decision-making. In addition, by integrating pathological classification, pathogen distribution, and organism-specific susceptibility profiles—including a formal comparison of ESBL-producing vs. non-ESBL-producing isolates—the study provides a clinically coherent framework for linking microbiological findings to therapeutic implications.

### 4.5 Limitations

Several limitations should also be acknowledged. First, this was a single-center study with a relatively small sample size, limiting the generalizability of the findings. Second, the analysis was primarily descriptive, and the absence of complete subgroup comparisons reduced the ability to quantify associations between disease severity, ESBL phenotype, and antimicrobial resistance patterns.

Third, anaerobic organisms were not comprehensively characterized, which may have resulted in an incomplete representation of the appendiceal microbiota. The use of swab-collected purulent material and the absence of anaerobic transport media may have further affected organism recovery, particularly for fastidious or anaerobic bacteria. The overwhelming predominance of Gram-negative isolates should therefore be interpreted with caution, as it likely reflects, at least in part, the methodological constraints of aerobic culture rather than the true biological distribution.

Fourth, all patients received preoperative antibiotics as part of the standardized perioperative protocol. Although the timing (30–60 min before incision) was designed to minimize the impact on intraoperative specimen collection, we cannot entirely exclude the possibility that antibiotic exposure influenced culture recovery or susceptibility patterns. The subset of patients who had received additional outpatient antibiotics before admission represents a potential confounder that warrants further investigation in larger studies.

Finally, clinical outcomes such as postoperative abscess formation, duration of hospitalization, antibiotic modification, and treatment failure were not analyzed, limiting the ability to connect microbiological findings with direct prognostic impact.

### 4.6 Future directions

Future studies should expand on the framework established here by incorporating multicenter cohorts, larger sample sizes, dedicated anaerobic culture or molecular microbiological techniques (e.g., 16S rRNA sequencing), and outcome-oriented analyses (19, 20). Formal prospective validation of the ESBL-stratified resistance patterns in a larger pediatric population would substantially strengthen the evidence base for risk-adapted empirical therapy in pediatric appendicitis.

## 5 Conclusions

In this cohort of pediatric patients with acute appendicitis, intraoperative purulent specimens yielded a high rate of pathogen recovery, with a marked predominance of Gram-negative bacteria. *E. coli* was the principal pathogen, and a substantial proportion of isolates were ESBL producers. These findings should be interpreted in the context of the study's aerobic culture methodology, which may have limited the recovery of anaerobic organisms.

The findings support the need for local microbiological surveillance and suggest that empirical antibiotic selection in complicated appendicitis should be informed by regional resistance patterns rather than relying solely on conventional historical regimens. A stratified approach—incorporating disease severity, local ESBL prevalence, and patient-level risk factors—is recommended for evidence-based empirical therapy.

## Data Availability

The raw data supporting the conclusions of this article will be made available by the authors, without undue reservation.
